# NGAL as Biomarker of Clinical and Subclinical Damage of Kidney Function after Coronary Angiography

**DOI:** 10.3390/diagnostics13061180

**Published:** 2023-03-20

**Authors:** Iliyana Petrova, Alexander Alexandrov, Georgi Vladimirov, Hristo Mateev, Ivaylo Bogov, Iva Paskaleva, Nina Gotcheva

**Affiliations:** 1 Clinic of Cardiology, National Heart Hospital, 65 Konioviza Str., 1309 Sofia, Bulgaria; 2 Central hospitalier Châlons-en-Champagne, 51 Rue du Commandant Derrien, 51000 Châlons-en-Champagne, France; 3 Laboratory Department, National Heart Hospital, 65 Konioviza Str., 1309 Sofia, Bulgaria

**Keywords:** contrast-induced acute kidney injury (CI-AKI), subclinical CI-AKI, NGAL, contrast media, coronary angiography

## Abstract

Contrast-induced acute kidney injury (CI-AKI) is a serious complication after angiographic examinations in cardiology. Diagnosis may be delayed based on standard serum creatinine, and subclinical forms of kidney damage may not be detected at all. In our study, we investigate the clinical use in these directions of a “damage”-type biomarker—neutrophil gelatinase-associated lipocalin (NGAL). Among patients with a high-risk profile undergoing scheduled coronary angiography and/or angioplasty, plasma NGAL was determined at baseline and at 4th and 24th h after contrast administration. In the CI-AKI group, NGAL increased significantly at the 4th hour (Me 109.3 (IQR 92.1–148.7) ng/mL versus 97.6 (IQR 69.4–127.0) ng/mL, *p* = 0.006) and at the 24th hour (Me 131.0 (IQR 81.1–240.8) ng/mL, *p* = 0.008). In patients with subclinical CI-AKI, NGAL also increased significantly at the 4th hour (Me 94.0 (IQR 75.5–148.2) ng/mL, *p* = 0.002) and reached levels close to those in patients with CI-AKI. Unlike the new biomarker, however, serum creatinine did not change significantly in this group. The diagnostic power of NGAL is extremely good—AUC 0.847 (95% CI: 0.677–1.000; *p* = 0.001) in CI-AKI and AUC 0.731 (95% CI: 0.539–0.924; *p* = 0.024) in subclinical CI-AKI. NGAL may be a reliable biomarker for the early diagnosis of clinical and subclinical forms of renal injury after contrast angiographic studies.

## 1. Introduction

With the increase in cardiovascular diseases worldwide, the relevance of contrast radiographic examinations as coronary or periphery angiographies is constantly growing as they provide valuable diagnostic and therapeutic management opportunities. One of the complications after the administration of a contrast agent is the early deterioration of kidney function. The term “contrast-induced nephropathy (CIN)” was introduced in 1999 by the European Society of Urogenital Radiology (ESUR) and was defined as the elevation of serum creatinine (SCr), with more than 0.5 mg/dL (44.2 µmol/L) or over 25% observed up to three days after the intravascular administration of contrast media in the absence of another etiologic cause [1]. Later, KDIGO introduced the AKIN criteria and the term “contrast-induced acute kidney injury (CI-AKI)”, emphasizing that it should not be differentiated from other forms of acute kidney injury (AKI). There was accepted a definition of CI-AKI as increased serum creatinine > 0.3 mg/dL (26.5 µmol/L) or its relative elevation by 1.5–1.9 times (≥50%) as compared to its baseline values [2].

Despite the tendency to unify these definitions in the present literature [3,4], the term “contrast-induced nephropathy” or the corresponding reference limit of SCr still prevails in previously published studies regarding angiographic studies in cardiology.

The frequency of CI-AKI after coronary intervention varies between 11.3% and 14.5% [5], but it can increase in special situations (such as acute myocardial infarction (MI) and chronic kidney disease (CKD)) to 36.9% [6]. A relatively high frequency of CI-AKI is also reported after transcatheter aortic valve implantation (TAVI)—22%—which may be important for the long-term prognosis of the intervention [7,8]. In patients with peripheral vascular disease, the frequency of CI-AKI is 11%, but it can reach 40.84% in cases with acute ischemia of the lower extremities due to severe comorbidity [9].

Although CI-AKI can sometimes be a transient condition, with the normalization of renal function within 7–10 days, in a number of patients, its occurrence can have unpredictable and serious consequences. According to data from the American National Cardiovascular Data Registry (NCDR), in patients with AKI after percutaneous coronary intervention (PCI), the in-hospital rates of MI, bleeding, and death were 3.8%, 6.4%, and 9.6%, respectively, compared with 2.1%, 1.4%, and 0.5%, respectively, in patients with no AKI reported. The rates of these events were the highest in the patients with AKI demanding dialysis [6]. The long-term consequences are also not to be underestimated, leading to an increased incidence of major adverse cardiac and cerebrovascular events among patients with CI-AKI within eight years of follow-up (54% vs. 15% vs. without CI-AKI) [10]. Additionally, a number of studies indicate that it may also have a long-term effect on kidney function [10,11]. According to the Alberta Registry [11], the proportion of patients with a sustained loss of kidney function three months after coronary angiography was 5.9% of patients without AKI, 28.2% of patients with mild AKI, and 59.1% among patients with moderate or severe AKI. According to other authors, the progressive deterioration of renal function within five years may be accelerated and more severe in patients with CI-AKI [10].

Assuming these consequences and the lack of specific therapy for CI-AKI, the main efforts of clinicians are focused on various preventive measures and timely diagnostic strategies. The elevation of the standard biomarker of kidney function serum creatinine, influenced by various external and internal factors, can be registered only when a new steady-state condition is reached, which can take different lengths of time after the impact of the damaging factor. Additionally, SCr lacks the kinetic properties necessary for real-time measurements of kidney dysfunction, the sensitivity to diagnose tubular injury before excretory failure develops, and the specificity to distinguish tubular injury from other causes for the elevated SCr level [12]. These considerations turn it into a delayed and insensitive marker of renal dysfunction.

On the other hand, in the last 10 years, the existence of a number of molecules such as KIM-1 (Kidney Injury Molecule-1), NGAL (Neutrophil Gelatinase-Associated Lipocalin), L-FABP (Liver Fatty Acid Binding Protein), and others, of which production increases rapidly after acute ischemic/toxic damage to the renal tubules, has been demonstrated [13,14,15,16,17]. Accumulating evidence for the application of new biomarkers and their ability to identify multiple additional processes in renal structures has led to the introduction of a new conceptual framework for AKI and their classification as “damage biomarkers” [18,19]. In comparison to standard “functional biomarker” serum creatinine, their main advantage is the distinction of subclinical forms of kidney injury and the earlier diagnosis of acute kidney damage.

In the field of interventional cardiology, where patients are usually discharged the day after contrast angiography, the pointed limitations of SCr may lead to the delayed or miss-diagnosis of CI-AKI. On the other hand, a number of clinical studies focused on NGAL [17,20] and KIM-1 [20,21], as well as some newer molecules such as Midkine [22] and micro-RNAs [23], have demonstrated early elevation within four to six hours after contrast administration. Elevation of cystatin C >10% at the 24th hour after exposure can predict an increase in serum creatinine >0.3 mg/dL [24] and reliably distinguish patients with CI-AKI [25]. L-FABP [26] and IL-18 [27,28] also were reported to increase before the 12th hour after angiography, but some authors have mentioned controversial results of limited specificity [20,29,30].

NGAL, as one of the more reliable early biomarkers, has been widely studied not only in terms of contrast kidney damage but also in other conditions such as critically ill patients in intensive care units or AKI after aortocoronary bypass surgery, with demonstrated good diagnostic power in individual studies [31,32,33] and some meta-analyses [34,35,36]. An important role in congenital kidney disease is demonstrated by the meta-analysis focused on urinary biomarkers in congenital hydronephrosis secondary to pelvic–ureteric junction obstruction. There are reported significantly higher levels of NGAL in this specific population [37].

The valuable role of new biomarker” is complemented by another advantage—the diagnosis of subclinical forms of kidney damage. Substitution of SCr in the definition of “CIN” with the same change in cystatin C (>25% increase from baseline levels) leads to the diagnosis of twice as many cases with contrast-induced renal damage—16.52% versus 37.19% [38]. Additionally, some authors reported that the “NGAL (+)/serum creatinine (−)” result in critically ill patients is an independent predictor of adverse outcomes regardless of the presence or absence of functional impairment [39].

Focusing on invasive cardiology, there is still no consistent and clear definition of subclinical CI-AKI, and no reliable single structural biomarker has been identified yet. Additionally, there is no available specific description of the dynamic changes after contrast administration. Active comparison of biomarkers between patients developing CI-AKI and subclinical forms of kidney damage is not yet properly addressed.

In that context, our aim is to investigate the diagnostic power of an established structural biomarker—NGAL for the early diagnosis of CI-AKI and the subclinical form of CI-AKI—among patients undergoing a scheduled coronary angiography.

## 2. Materials and Methods

### 2.1. Study Populations

The investigation of the diagnostic significance of NGAL was conducted in a sample including a total of 45 patients. Inclusion criteria were the presence of a high cardiovascular risk profile (defined by arterial hypertension, diabetes mellitus type 2 (DM), metabolic syndrome, and/or dyslipidemia), coronary artery disease (defined by a history of myocardial infarction, previous percutaneous coronary intervention, or aortocoronary bypass (ACB) revascularization), and preserved kidney function (GFR ≥ 60 mL/min/1.73 m^2^, calculated according to the MDRD (Modification of Diet in Renal Disease) formula). In the study, we selected only patients with stable angina and stable clinical conditions undergoing elective coronary angiography with/without percutaneous angioplasty.

The main exclusion criteria were different forms of acute coronary syndrome (acute myocardial infarction with and without ST–elevation (STEMI, NSTEMI)), hemodynamic instability, or cardiogenic shock; the need for emergency cardiac or other surgical intervention; advanced and decompensated chronic congestive heart failure (NYHA functional class IV); established left ventricular (LV) ejection fraction (EF) ≤35% by echocardiographic measurement; history of AKI in the last month; moderate CKD (30–60 mL/min/1.73 m^2^) and end-stage CKD with renal-replacement therapy; the presence of liver dysfunction; neoplasm; clinical and laboratory data on acute inflammatory disease.

All patients underwent an invasive angiographic examination using the same low-osmolar contrast agent—Iomeprol. For the prevention of contrast-induced nephropathy, active hydration with normal saline solution was carried out in a dose according to body weight and current clinical condition, as assessed by the attending physician. All participants provided written informed consent to participate before coronary angiography and blood sampling. The study protocol was reviewed and approved by the appropriate institutional review board (National Heart Hospital Ethics Committee; Protocol No. 11/04.12.2013).

### 2.2. Laboratory Measures

According to the study design, blood samples for serum creatinine and plasma NGAL were obtained from all selected patients as follows: on the day before the angiographic examination (designated as baseline), at the 4th hour, and at the 24th hour after the patient’s return from the catheterization laboratory (defined as samples at the corresponding hour after the contrast administration). The laboratory measurement of serum creatinine was made according to the Jaffe method, and NGAL was determined with a turbidimetric method and a reagent kit NGAL Test^TM^ from BioPorto Diagnostics A/S (Denmark, Copenhagen), applied to an Olympus 400 biochemical analyzer.

### 2.3. Study Endpoints and Definitions

The main objective of the study is the development of contrast-induced acute kidney injury after angiographic examination, defined as an absolute (≥44 μmol/L) or a relative (≥25%) increase of serum creatinine from baseline or a 25% decrease in glomerular filtration rate (calculated by the MDRD formula) up to 48 h after contrast administration. The patients who met these criteria were classified as the group with CI-AKI. The subclinical form of CI-AKI was accepted in patients without specific dynamics of serum creatinine but a documented increase of plasma NGAL ≥25% from baseline values. The rest of the patients with no CI-AKI or a subclinical form of CI-AKI served as the control group.

### 2.4. Statistical Methods (Statistical Analysis)

We used SPSS (Statistical Package for the Social Sciences) version 16.0. for data processing in the study. The continuous variables are presented as average values ±standard deviation (SD) or the median (Me) and interquartile range (IQR), where this is necessary. For the estimation of dependencies between the descriptive categorical variables, we applied the Pearson chi-square test. When more than 20% of the cells in the table on conjugation had expected frequencies lower than 5 and/or if there was an expected frequency less than 1 in any cell, we applied Fisher‘s exact test. The one-sample Kolmogorov–Smirnov test and the Shapiro–Wilk test were used for the estimation of the form of frequency distribution relative to the form of normal distribution.

For the comparison of rank data between more than two independent groups, when the shape of the frequency distribution differs from normal, the nonparametric Kruskal–Wallis test was applied. The comparison of repeated measurements on the continuous variables of serum creatinine, GFR, and NGAL was performed using the nonparametric Wilcoxon signed ranks test.

To investigate the equality between the average values of more than two groups, the test for one-factor dispersion analysis (analysis of variance—ANOVA) was applied. Immediately after it, in order to establish significance and conduct multiple comparisons, Tukey post hoc tests were applied. When verifying a form with a normal distribution of the studied variable in the individual groups, with a view to conducting a comparative pairwise analysis, *t*-tests for two independent groups (independent-samples *t*-test) or *t*-tests for two dependent groups (paired-samples *t*-test) were applied. The presence of a frequency distribution of the studied indicator with a shape different from the normal one necessitated the application of the nonparametric Mann–Whitney test when comparing two independent groups.

The diagnostic ability of the studied parameters (creatinine, GFR, and NGAL) was assessed by applying ROC analysis (receiver operating characteristic) and calculating the area under the curve (AUC). A value of AUC of 0.5 matches the lottery, whereas the value of 1.0 is relevant to a perfect biomarker. The used critical level of significance is α = 0.05, and the corresponding zero hypothesis is true when the *p*-value is smaller than α.

## 3. Results

Among the 45 patients in the study, CI-AKI was diagnosed in 12 (26.7%) patients (CI-AKI group). The subclinical CI-AKI group included 15 (33.3%) patients, and the control group was formed by 18 (40%) patients.

The risk profile of the total sample was defined by arterial hypertension (*n* = 45/100%), dyslipidemia (*n* = 44/97.8%), diabetes mellitus type 2 (*n* = 41/91.1%), overweight (BMI 25–29.9 kg/m^2^ at *n* = 17/37.8%), and obesity (BMI ≥ 30 kg/m^2^ in *n* = 21/46.7%). The distribution between the individual groups ais presented in Table 1, with no significant differences reported between them.

The main indication for the angiographic examination in the studied cohort of patients was stable angina pectoris (*n* = 39/86.6%), and some of them have a history of myocardial infarction (*n* = 17/37.8%). Previous percutaneous coronary intervention (PCI) (*n* = 15/33.3%) or operative coronary revascularization (*n* = 7/15.6%) and heart failure II–III by NYHA class (*n* = 13/28.9%) also contributed to the cardiac profile of the sample. Diagnostic angiography was performed in 48.89% of all selected patients, and in 51.11%, it was necessary to switch to one-stage PCI. As evident from Table 1, in patients with CI-AKI, there was a trend for the predominance of multivessel coronary disease and a significantly higher frequency of previous aortocoronary bypass revascularization. The latter had an essential role in determining the need for the application of a larger amount of contrast media for the angiographic visualization of anatomy among such patients.

The evaluation of renal function was carried out by the simultaneous measurement of serum creatinine and plasma NGAL in a series of blood samples obtained at baseline before and at the 4th and 24th hour after the coronary angiography. For the diagnosis of CI-AKI, creatinine was monitored until the 48th hour after the contrast administration. Detailed information on the values of these indicators, presented as the median and interquartile range (IQR) in the different patient groups, is shown in Table 2.

In the control group of patients, serum creatinine and corresponding GFR remained unchanged after the contrast administration compared to baseline levels (Table 2). A similar trend was registered with measurement of the new biomarker NGAL—values reported at baseline (Me 80.3 (66.9–86.2) ng/mL), at the 4th hour (Me 76.6 (65.3–87.6) ng/mL) and at the 24th hour (Me 78.0 (66.4–88.5) ng/mL) were extremely close (*p* = 0.943; *p* = 0.653 respectively).

**Figure 1:**
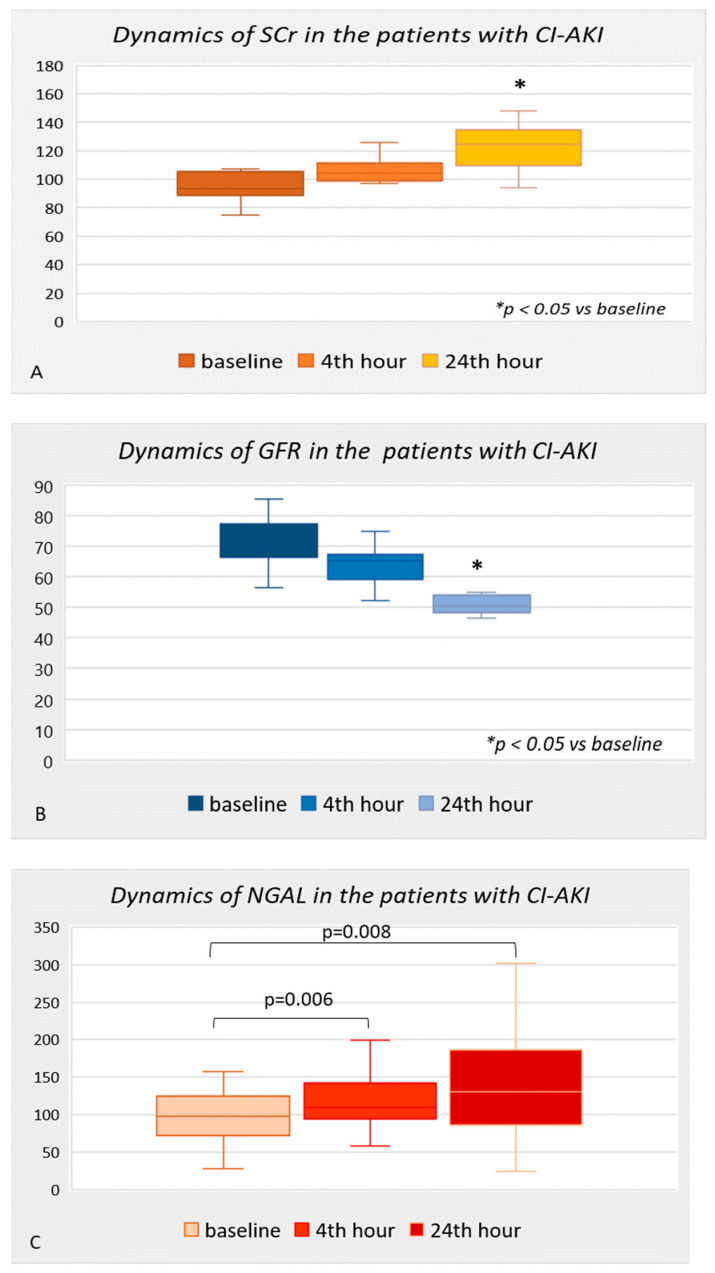
Dynamic changes in all biomarkers in the group of patients with contrast-induced acute kidney injury (CI-AKI). Panel (**A**)—dynamics of serum creatinine; Panel (**B**)—dynamics of GFR; Panel (**C**)—dynamics of NGAL; *—comparison of biomarkers at 24th hour versus baseline values. SCr—serum creatinine (μmol/L); GFR—glomerular filtration rate (mL/min/1.73 m^2^); NGAL—neutrophil gelatinase associated lipocalin (ng/mL).

In patients with the development of CI-AKI, dynamic changes were found in all the investigated biomarkers (Figure 1). Serum creatinine increased from baseline levels as the median (93.5 (88.3–105.9) µmol/L) in this group 24 h after the contrast angiography (124.5 (108.5–136.3) µmol/L; *p* = 0.002) and remained high until the 48th hour (106.0 (91.8–119.5) µmol/L; *p* = 0.007). The change in GFR follows the same trend, but in the opposite direction—compared to the initial levels (69.0 (66.0–78.2) mL/min), a significant decrease was reported at the 24th hour (50.5 (47.7–54.5) mL/min, *p* = 0.002). The laboratory measurements of plasma NGAL in this group of patients showed that the median (IQR) initial value was 97.6 (69.4–127.0) ng/mL, but it already increased very quickly at the 4th hour after the contrast administration to 109.3 (92.1–148.7) ng/mL (*p* = 0.006). A significant increase in the biomarker continued at the 24th hour, where the reported levels were even higher and reached an average of 131.0 (81.1–240.8) ng/mL (*p* = 0.008) (Figure 1C).

**Figure 2:**
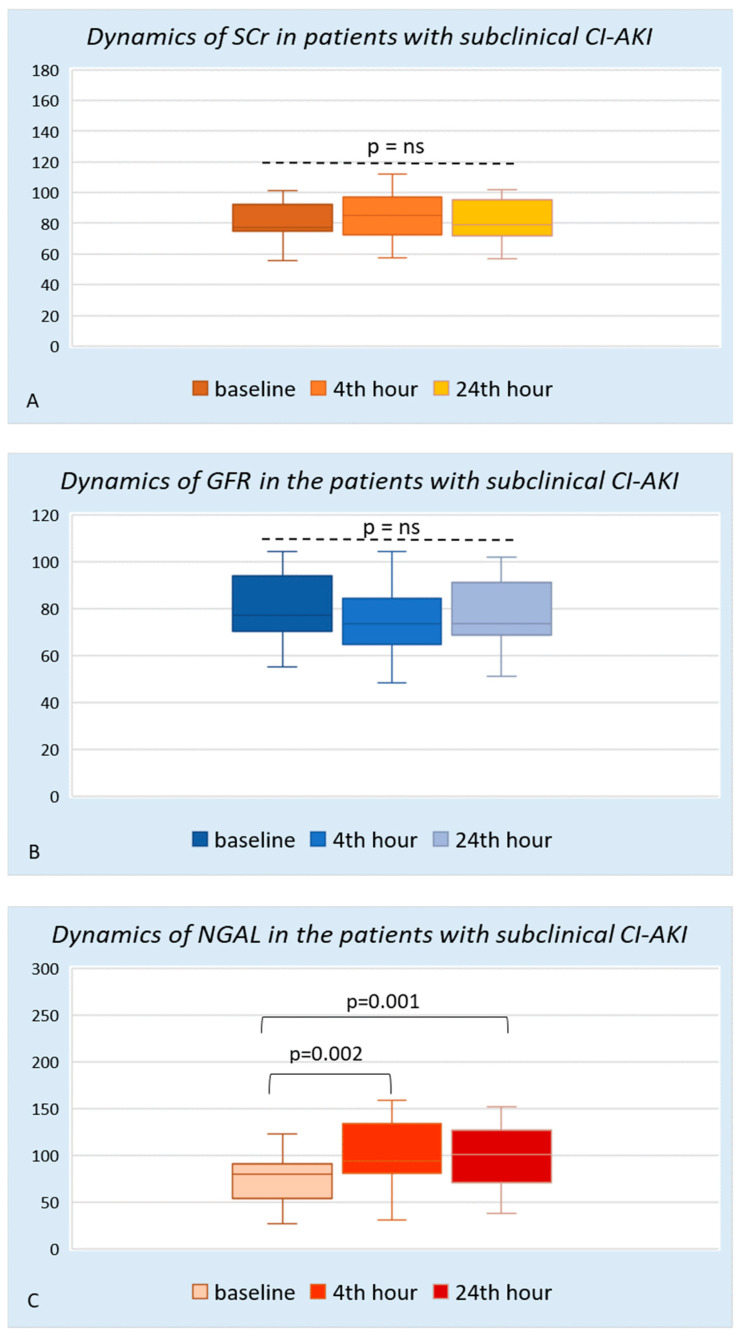
Dynamic changes of all biomarkers in the group of patients with subclinical contras-induced acute kidney injury (CI-AKI). Panel (**A**)—dynamics of serum creatinine; Panel (**B**)—dynamics of GFR; Panel (**C**)—dynamics of NGAL. SCr—serum creatinine (μmol/L); GFR—glomerular filtration rate (mL/min/1.73 m^2^); NGAL—neutrophil gelatinase associated lipocalin (ng/mL). ns—non significant.

In the group with subclinical CI-AKI, the standard marker of renal function, serum creatinine, maintained its levels and was almost unchanged at the 24th hour (Me 79.0 (71–96) μmol/L, *p* = 0.292) and the 48th hour (Me 76.5 (69.5–89.5) μmol/L, *p* = 0.889) compared to the baseline values (Figure 2A). GFR calculated for the corresponding time intervals also did not show a significant change (Table 2). However, the changes in plasma NGAL among this group of patients are interesting. Compared to the baseline values presented as the median (IQR 80.0 (44.4–94.1) ng/mL), already on the 4th hour after the end of the angiographic examination, a strong increase in the biomarker was registered, with a level of 94.0 (75.5–148.2) ng/mL (*p* = 0.002). At the 24th hour after the examination, increased values compared to the baseline (100.7 (55–132.2) ng/mL, *p* = 0.001) remained significantly higher (Figure 2C).

**Figure 3:**
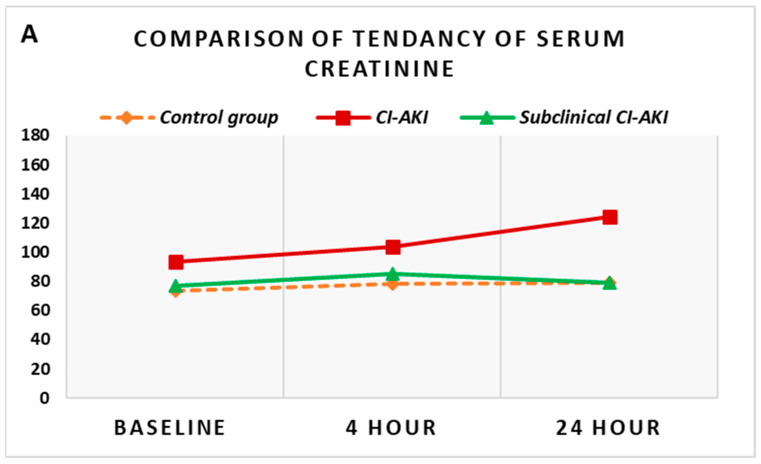

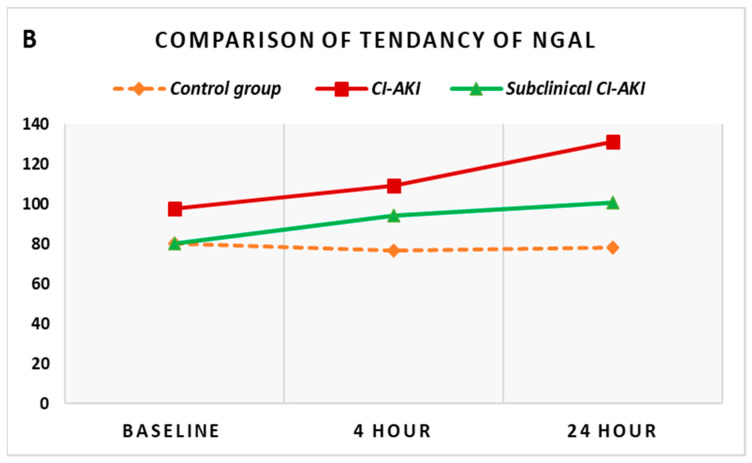
Comparison of dynamic changes in serum creatinine (**A**) and plasma NGAL (**B**) in the different groups. Control group versus CI-AKI group—creatinine—baseline (*p* = 0.005), 4th hour (*p* < 0.001), 24th hour (*p* < 0.001); NGAL—baseline (*p* = 0.072), 4th hour (*p* = 0.001), 24th hour (*p* = 0.005); control group versus group of subclinical CI-AKI—creatinine—baseline (*p* = 0.625), 4th hour (*p* = 0.458), 24th hour (*p* = 0.928); NGAL—baseline (*p* = 0.899), 4th hour (*p* = 0.024), 24th hour (*p* = 0.086); CI-AKI versus subclinical CI-AKI—creatinine—baseline (*p* < 0.05), 4th hour (*p* < 0.05), 24th hour (*p* < 0.05); NGAL—baseline (*p* = 0.088), 4th hour (*p* = 0.354), 24th hour (*p* = 0.164). CI-AKI (contrast-induced acute kidney injury).

Moreover, we conducted an additional analysis focused on the comparison of each indicator in the same time interval in the different groups. Standard markers of renal function (serum creatinine and GFR) in patients who developed CI-AKI were significantly higher compared to the control group of patients (Figure 3A). In the same comparison, the baseline levels of NGAL did not differ significantly between the two groups, but with the onset of acute kidney injury and the increase of NGAL, the levels reached at the 4th and 24th hours after the contrast administration were significantly higher compared to the controls (*p* < 0.005) (Figure 3B).

The comparative analysis of the group with subclinical CI-AKI was carried out against the other two groups—the control group and the group with CI-AKI. The comparison with the control group demonstrated no significant difference between all reported values of serum creatinine and GFR (Figure 3A). The increase in NGAL, however, which was observed in the group with subclinical CI-AKI, was significantly higher at the 4th hour compared to controls (*p* = 0.024) (Figure 3B). The comparison of the groups with CI-AKI and subclinical CI-AKI shows that while all the values of serum creatinine and GFR were significantly different between the two groups (*p* < 0.05), the initial levels of NGAL and the subsequently monitored dynamic levels were extremely close, and no statistically significant difference was found between them. The simultaneous general presentation of these trends for the three groups can be found in Figure 3.

**Figure 4:**
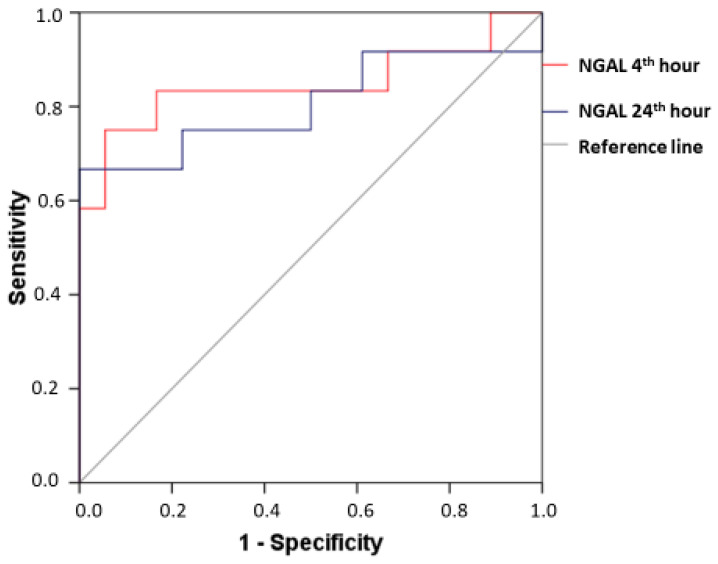
ROC curves for the diagnostic ability of plasma NGAL, measured at the 4th and 24th hour after the angiographic examination in the CI-AKI group.

**Figure 5:**
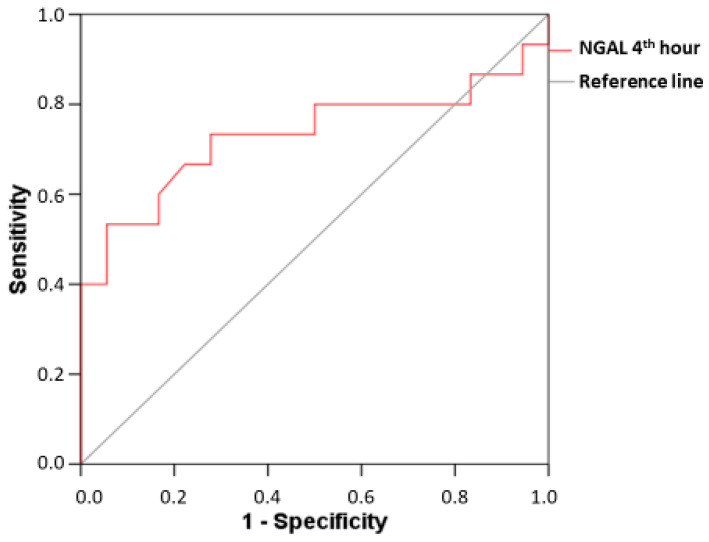
ROC curve for the diagnostic ability of plasma NGAL, measured at the 4th hour after the angiographic examination in the group of subclinical CI-AKI.

ROC analysis provides a clear insight into the diagnostic value of the new biomarker. In patients with CI-AKI, the plasma NGAL at the 4th hour after the contrast administration demonstrated AUC 0.847 (95% CI: 0.677–1.000; *p* = 0.001), sensitivity 83.33%, and specificity 83.33% at a cut-off value of 90.20 ng/mL. The diagnostic power remained significant, considering the changes at the 24th hour after the angiographic examination—AUC 0.806 (95% CI: 0.617–0.994; *p* = 0.005) with a sensitivity of 75%, a specificity of 77.78%, and a cut-off value of NGAL 88.30 ng/mL (Figure 4). In patients with a subclinical form of CI-AKI, the good diagnostic prediction of NGAL was preserved at the 4th hour after the angiographic examination—AUC 0.731 (95% CI: 0.539–0.924; *p* = 0.024), with sensitivity 73.33% and specificity 72.22% at a cut-off value of 85.65 ng/mL (Figure 5).

## 4. Discussion

In our study, the follow-up of renal function with the standard biomarker serum creatinine and the new biomarker NGAL showed that in the patients of the CI-AKI group, all indicators changed significantly after the administration of a contrast media. Creatinine peak values were reached at the 24th hour, and the reported glomerular filtration rate in this hour range was correspondingly the lowest. Plasma NGAL demonstrated a significant increase at 4th and 24th hour from baseline, but the statistical analysis indicated that the majority of the biomarker increase occurred in the first few hours after contrast administration.

Contrast-induced acute kidney injury (or contrast-induced nephropathy, as used in the past) continues to be a serious problem after diagnostic and therapeutic contrast-enhanced angiography, ranking third as a cause of hospital-acquired acute renal failure [40]. As a result of the implementation of various preventive strategies in the last decade, a tendency to reduce the incidence of CI-AKI has been reported [41,42]. On the other hand, a number of authors have emphasized that intra-arterial (ia) administration of contrast media through a catheter during angiography, with or without percutaneous coronary intervention, is associated with a higher incidence of post-contrast AKI than intravenous (iv) administration [43,44]. While some literature data have reported rates of CI-AKI after coronary interventions in the general population between 11.3% and 14.5% [5], other authors emphasize that the combination of more risk factors leads to a significant increase in its frequency—from 26.6% up to 36.9% [6].

In our study, patients with a high risk profile (arterial hypertension, dyslipidemia, DM type 2 in >90% of patients) and a high ischemic burden with clinically manifested or proven coronary heart disease (stable angina pectoris > 85%, history of MI, previous PCI or ACB revascularization > 48% of all patients) were enrolled. The frequency of CI-AKI was found to be 26.7%, which is close to the 24.1% reported in the literature in a cohort of patients with type 2 diabetes mellitus [45].

NGAL is a small protein that is normally expressed in healthy individuals in very small amounts in various types of cells in the body [46]. Immediately after the occurrence of acute kidney injury, the production of NGAL is increased in the distal parts of the nephron, the thick ascending part of the Henley’s loop, the distal tubules, and the collecting ducts [47,48], which results in increased urinary and plasma NGAL levels due to increased secretion from the apical and basolateral surface of the nephron epithelium. Experimental studies have shown that plasma NGAL increases as a result of the “backflow” of increased synthesis molecules to the systemic circulation [49]. Some authors emphasize that AKI leads to a dramatic increase in RNA expression for NGAL in distant organs [49] such as the liver and lungs. The overexpressed protein, released into the circulation, forms a distant systemic pool that is the source of the plasma levels of NGAL. In the hope that “renal troponin” may have been discovered, a number of authors have focused on it in various clinical situations—contrast-induced nephropathy, critically ill patients in intensive care units, patients after cardiac surgery, and others. Special attention is paid to cohorts of patients with chronic kidney disease, where some authors have reported specific biomarker kinetics and higher baseline NGAL concentrations [50,51,52].

In this direction, several studies in the literature have reported the clinical use of NGAL among patients with coronary heart disease and baseline-preserved renal function who underwent elective angiography [17,31,32,33,53,54]. As Bachorzewska-Gajewska [32] demonstrated in one of the first studies, which a number of other authors [31,33,55] subsequently confirmed, NGAL significantly increases in the first hours after contrast administration. In the study by Shaker et al. [33], it was demonstrated that compared to the baseline values of plasma NGAL of 52.5 ± 13.8 ng/mL, at the 4th hour after angiographic examination, an increase to 88.5 ± 16.4 ng/mL was reported (*p* < 0.001); at the 24th hour, the values were 63.6 ± 10.5 ng/mL (*p* < 0.001). The regression analysis proved a positive significant correlation between the levels of the new biomarker and serum creatinine in each of the studied time intervals. According to Padhy et al. [31], serum NGAL is a biomarker with a “narrow diagnostic window” in which peak values can be reached within 4 h after contrast angiography examination and remain significantly higher for up to 24 h but, by 48 h, can be completely normalized. Liao et al. [17] reported in their study of 240 patients that the diagnostic power of serum NGAL was extremely good—at six hours after contrast examination, the area under the curve (AUC) was 0.81 (*p* = 0.03), with a sensitivity of 97.64% and a specificity of 67.78% at a cut-off point of 96.35 ng/mL; at the 24th hour, the AUC was 0.89 (*p* < 0.01), and sensitivity 96.63% and specificity 68.72% were at the established reference level of 97.57 ng/mL. In summary, NGAL is emerging as an early biomarker for contrast-induced AKI, with a significant increase within 24 h after the procedure [17,27,32,33,54], significantly rising before the rising of the standard serum creatinine, which has significantly different values that are registered only on the second day [32,54].

The results of our study fully confirm dynamic changes in NGAL and prove its good diagnostic value as an early marker for the onset of CI-AKI, considering the data from the ROC analysis (at 4th hour—AUC 0.847, cut-off point of plasma NGAL 90.20 ng/mL, sensitivity 83.33%, specificity of 83.33%). These results fully correlate with those cited in the literature (AUC 0.81–1.00 at 4–6 h [18,42] and AUC 0.89 at 24th hour [42]) and presented in several meta-analyses focused on CI-AKI [34,35,36].

It should be noted, however, that the increase in serum creatinine within the first 24 h after angiography is somewhat different from the trends described in some studies [32,50,55]. This may be due to design differences across studies. In many study designs, it is assumed that the laboratory samples were obtained at different times, with creatinine being reported only at baseline and at the end of the observed period (within 48–72 h after the procedure) [32,50,55]. In contrast, we examined both biomarkers from blood samples obtained simultaneously at the same time interval, allowing a full parallel comparison of their dynamic changes. Furthermore, our selected cohort of patients with predominant diabetes mellitus type 2 (91.1%) was completely different from most studies [17,27,31,32,54,55,56,57], where the incidence of DM was between 14% and 34%. From this perspective, the only study reporting the role of NGAL exclusively in diabetic patients is presented by Ashalatha et al. [45], and its results showed that serum creatinine and NGAL increased significantly as early as the 4th hour, and a difference (when compared to the “non-CIN” group) was observed at the 24th hour, but only for creatinine. The authors concluded that patients with diabetes mellitus and preserved renal function are more likely to suffer from subclinical renal impairment, considering them much more susceptible to contrast damage, leading to the early elevation of biomarkers. They assumed that the dynamic changes of indicators may be different in diabetic patients and in individuals without DM [45].

This direction of scientific reasoning once again emphasizes the importance of a comprehensive time-wise study of kidney dysfunction in a completely different aspect—the existence, diagnosis, and prognosis of subclinical forms of damage. As already mentioned, the modern understanding of AKI emphasizes a diagnostic approach based on the simultaneous measurement of functional and structural biomarkers [18,19]. Based on this concept, patients undergoing invasive procedures with contrast administration should also be screened for the development of renal injury or impaired function by the evaluation of both types of biomarkers for AKI [58]. Although some authors propose the introduction of terms such as “CI-AKI with structural damage” or “CI-AKI with kidney dysfunction”, this distinction remains only theoretically grounded [58].

In the literature, there is no clear definition of subclinical CI-AKI or a clearly defined frequency of this group of events. Initially, some authors [45] only describe that among the group “without CIN”, there are individuals with an increase in NGAL similar to that reported in CIN. On the other hand, other studies [51,59] suggest a limit, such as an increase of >25% of the biomarker, or assume that for the diagnosis of subclinical AKI, the biomarker must increase by two times compared to its baseline levels [52,60]. In the studies of Breglia et al. [61] and Rozenfield et al. [62], an absolute value is introduced as a reference limit, above which the biomarker is reported as positive. While some authors have reported an incidence of subclinical AKI of 11.1% [52], others have reported a “biomarker (+)/creatinine (−)” cohort with an incidence of 32.6% [59] to 44% [62]. A meta-analysis [39] of 10 prospective studies among critically ill patients found that based on the “NGAL (+)/serum creatinine (−)” result, up to 40% more cases of AKI could be identified, which would have been missed if the standard creatinine-based AKI criteria are used. Our results are complementary to the sources reported in the literature regarding the frequency of this form of renal damage.

In our study, we accepted subclinical CI-AKI to be defined as an increase in plasma NGAL of >25%, which fully corresponds to the change in the biomarker registered in the group with the clinically manifested form of CI-AKI. In relation to the entire studied sample, this result was found in 33.3% of patients, which may exceed some of the cited sources [52] but covers the wide range reported by others [62]. As already noted, the selection of a cohort of patients with diabetes mellitus may have altered the sample profile, delivering a higher risk of subclinical renal impairment [45]. Monitoring of NGAL in such cases may help to detect AKI before the change in serum creatinine.

In the study by Alharazy et al. [51], among 100 patients undergoing elective coronary angiography ± PCI, the diagnostic role of serum NGAL and cystatin C in the detection of “CIN” was investigated. The authors reported that this event (defined as a >25% rise in serum creatinine by 48 h) occurred in 11% of patients (*n* = 11/100), and both biomarkers had good diagnostic performance at 24 h after the contrast angiography. It is interesting to note that a limit of 25% increase in serum NGAL was registered in *n* = 7/11 patients with “CIN” and in *n* = 12/87 patients in the “non-CIN” group. An elevation of cystatin C of >25% was found in only four of the “CIN” patients and among one of the remaining cohorts. The authors hypothesized that, in addition to diagnosing contrast-induced renal injury, the new biomarkers are likely to capture cases with subclinical AKI. The latter is only described as a finding, but no further analysis has been conducted in this direction. On the other hand, an assumption is made that such patients are likely to be missed in the standard diagnostics of CIN.

We have managed to advance further in our study by the inclusion of a group with subclinical CI-AKI, monitoring dynamic changes in biomarkers at all time intervals in parallel with the changes observed in a group with clinically manifested CI-AKI. It can be seen from the obtained results in the patients with subclinical CI-AKI that serum creatinine (respectively, GFR) had no significant changes compared to its initial levels through the entire observation period (48 h after the end of angiography). In terms of dynamics, however, plasma NGAL increased significantly as early as the 4th hour after the contrast administration and maintained its high levels until the 24th hour. The diagnostic power of the new biomarker for early detection of renal damage is relatively good, as shown by the ROC analysis, with AUC 0.731 (*p* = 0.024), sensitivity 73.33%, and specificity 72.22% at a cut-off value of 85.65 ng/mL.

The comparative analysis against the other two groups clearly shows the positioning of this cohort of patients in the whole sample. Compared to the control group, patients with subclinical CI-AKI were significantly distinguished only by the values of plasma NGAL, which increased early after the end of contrast angiography (at the 4th hour). Compared to the group with CI-AKI, a significant difference was found only in the dynamics of serum creatinine (respectively, GFR). Based on these data, we can conclude that the group with subclinical CI-AKI occupies an intermediate place in the clinical continuum between patients without renal function impairment and patients with clinically manifested contrast-induced nephropathy. In other words, if the classic creatinine-based definition of AKI is solely applied in clinical practice, the only patients who will differ from the entire study cohort are those with CI-AKI. Patients in the control group and those with an “NGAL+” result, having close values (*p* = NS) of serum creatinine levels that do not change over time after the contrast study, will be misidentified as individuals without impaired renal function.

The first study designed to specifically investigate subclinical AKI was reported by the Akrawinthawong group et al. [52] and was conducted among 63 patients, with GFR < 90 mL/min/1.73 m^2^, undergoing routine angiographic examination. CI-AKI was defined according to the AKIN criteria (>0.3 mg/dL or >50% increase in serum creatinine up to 48 h), while “subclinical AKI” was defined as an increase in serum NGAL ≥ 2 times compared to baseline levels, without a concurrent change in creatinine. According to the results, CI-AKI was diagnosed among *n* = 8/63 (12.7%) of the examined patients, while in seven others (*n* = 7/63; 11.1%), subclinical AKI was identified. A general examination of all patients with AKI (defined by AKIN and subclinical form) showed that 23.8% had certain renal dysfunction after the administration of contrast media, and in all of them, the peak values of NGAL were significantly higher than at baseline levels. The study confirms the fact that the application of one of the new structural biomarkers captures almost twice as many cases of AKI. However, the authors emphasize the need for more research in this direction.

Our study differs from the aforementioned one by Akrawinthawong et al. [52] in terms of the selected patient cohort, including patients with different levels of renal function (GFR is in a wide range, from 15 to 90 mL/min), which is also reflected in the different baseline levels of NGAL cited by the authors (360.29 ± 227.94 ng/mL at GFR 15–30 mL/min versus 114.02 ± 57.42 ng/mL at GFR 60–90 mL/min). Considering that the existence of chronic kidney disease affects the level of NGAL [50,63] as well as our preliminary data in this direction [64], we turned our attention to a more strictly selected sample, according to renal function, examining separately the characteristics of NGAL among patients with GFR values above 60 mL/min.

Breglia et al. [61] also reported the occurrence of CI-AKI and subclinical CI-AKI after intra-arterial administration of contrast material in patients with GFR > 60 mL/min, but they applied a combination of structural biomarkers urinary NGAL, IGFBP7 (insulin-like growth factor-binding protein 7), and TIMP-2 (tissue inhibitor of metalloproteinase-2), measured 4–8 h post-procedure. According to the study design, CI-AKI was defined according to the KDIGO criteria (creatinine >0.3 mg/dL or >50% compared to baseline levels), and subclinical CI-AKI is defined as a positive result for the relevant biomarker (TIMP-2 и IGFBP7 > 0.3 (ng/mL)^2^/1000; NGAL > 90 μg/L). Out of a total of 100 selected patients with performed neurological invasive procedures, none of them was found to have CI-AKI according to the KDIGO criteria. At the same time, the studied biomarkers were positive in 13 patients (the NGAL (+) result was found in 5 patients, and TIMP-2 and IGFBP7 (+) results in 10 patients; in 3 patients, all biomarkers were positive). The critical message from this study is that while in a low-risk patient population (assessed as such according to the Mehran score), no cases of CI-AKI defined by creatinine were observed; subclinical CI-AKI, defined as the elevation of biomarkers of the lesion, was found in >10% of patients with contrast diagnostic/therapeutic procedures.

The importance of subclinical forms of AKI is noted by a number of authors. Haase‘s meta-analysis demonstrated that the “NGAL (+)/serum creatinine (−)” patient cohort had an increased risk of prolonged ICU stay, initiation of renal-replacement therapy, and increased mortality [39]. According to other authors, patients with positive new biomarkers (urinary KIM-1 and urinary NGAL) compared to patients with negative results have an increased risk of renal-replacement therapy and in-hospital mortality [65,66]. A study of STEMI patients undergoing primary angioplasty reported that 44% of patients had an “NGAL(+)/creatinine(−)” result (defined as NGAL values ≥100 ng/mL), and 14% of patients had an “NGAL (+)/creatinine (+)” result. Compared to the “NGAL (−)/creatinine (−)” subgroup, NGAL positivity alone resulted in a significantly increased risk of adverse in-hospital events (46% vs. 64%, respectively; OR 2.1; 95% CI 1.1–4.5; *p* = 0.05) [62] Other authors [67] demonstrated that even the baseline measurement of higher NGAL values could be associated with an increased frequency of major adverse events and overall mortality among patients from the third quartile (143.9 to 567.9 ng/mL) compared to a group from the first quartile (25.4 to 83.7 ng/mL). Recent studies in the field of subclinical CI-AKI demonstrate, in addition to a higher frequency of these events when applying the NGAL-based definition (18%), the persistence of subclinical damage for more than one month among half of the patients [68].

In summary, our presented data contribute to the current scientific literature on the diagnosis of subclinical forms of acute kidney injury after contrast angiography, demonstrating the characteristics of renal biomarkers in a relatively homogeneous cohort of patients in terms of high-risk profiles and baseline renal function. A number of the conducted studies included mixed cohorts of patients and focused primarily on the application of NGAL as a diagnostic marker for a clinically apparent form of CI-AKI. An advantage of our study is the investigation with an equal focus on clinical and subclinical forms of AKI, applying the current ADQI recommendations and two different types of renal biomarkers.

### Limitations of the Study

The relatively small sample size should be noted. Nonetheless, both in the field of contrast nephropathy and in a number of other emerging fields related to imaging methods, relatively small cohorts with less than 100 patients have been reported [33,52]. The relatively narrow follow-up interval of the patients, up to 48 h after the contrast examination, did not allow us to establish whether some of the patients with subclinical forms of kidney damage did not demonstrate a delayed increase in serum creatinine. On the other hand, the definition we apply for CI-AKI is in accordance with the one adopted in the literature [1,4]. Considering that NGAL is described in a number of sources as a marker with a “narrow diagnostic window” [31] in the first hours after exposure to the damaging agent, we have fully justified the biomarker follow-up in our study to be as early as possible after the contrast administration procedure.

## 5. Conclusions

Contrast-induced acute kidney injury is a potentially serious complication after angiographic studies, with long-term consequences on renal function and overall survival. The lack of specific treatment, the unconvincing success of a number of preventive measures, and the complex involvement of multiple pathophysiological mechanisms determine the continued relevance of this pathology. The acceptance of CI-AKI as a transient benign disorder of renal function and the disadvantages of serum creatinine as a diagnostic marker can only lead to the underestimation of the problem. NGAL is a damage biomarker that can be successfully implemented in the diagnostic process by integrating modern ADQI recommendations. Our study has determined that the assessment of NGAL plasma levels among patients undergoing scheduled angiography should increase after the administration of a contrast agent, with good diagnostic power for the differentiation of both clinical and subclinical forms of CI-AKI. Using the structural biomarker enabled the identification of 33% more patients with renal impairment that would have been missed with the standard creatinine-based definition. Validation of the finding among a larger group of patients and a cohort with chronic kidney disease should be considered in future research.

## Figures and Tables

**Table 1:** The main clinical characteristics of the studied patients by groups.

Parameters	Control Group (*n* = 18)	CI-AKI Group (*n* = 12)	Group of Subclinical CI-AKI (*n* = 15)	*p*
Age (mean ± SD)	61.72 ± 7.71	62.9 ± 11.43	60.53 ± 6.73	0.012
Male gender (%)	44.4	83.3	46.7	0.366
Arterial hypertension (%)	100	100	100	1.000
Diabetus mellitus Type 2 (%)	88.9	100	86.7	0.837
Stable angina (%)	77.8	91.7	93.3	0.730
History of myocardial infarction (%)	50	25	33.3	0.740
Previous PCI (%)	33.3	25	40	0.504
Previous aortocoronary bypass surgery (%)	11.1	41.7 *	0	0.020
Baseline serum creatinine (μmol/L) [Me(IQR)]	73.6 (67–95)	93.5 (88.3–105.9)	77.0 (74–92)	<0.05
Baseline GFR (mL/min/1.73 m^2^) [Me(IQR)]	79.8 (72.4–87.3)	69.0 (66.0–78.2)	77.1 (68.8–94.3)	<0.05
LV ejection fraction (%)	55.32 ± 8.87	55.17 ± 8.33	53.53 ± 9.73	0.640
**Coronary artery disease (CAD) type**				
One-vessel disease (%)	27.8	16.7	6.7	0.133
Two vessel disease (%)	11.1	25	33.3	
Multivessel disease (%)	22.2	50	26.7	
Diagnostic coronary angiography and one-stage PCI (%)	44.4	66.7	46.7	0.695
Volume of contrast medium (mL)	143.0 ± 104.7	204.08 ± 165.00	164.53 ± 120.44	0.432
Drug antidiabetic therapy (%)	61.1	83.3	46.7	0.142
Metformin intake (%)	38.9	58.3	40	0.142
Concomitant therapy with ACE inhibitor (%)	61.1	41.7	46.7	0.546

PCI—percutaneous coronary intervention; LV—left ventricular; ACE—angiotensin convertase enzyme inhibitor; Me—median; IQR—interquartile range. * *p* < 0.05 when compared to a group without aortocoronary bypass surgery (subclinical CI-AKI).

**Table 2:** Presentation of serum creatinine, glomerular filtration, and NGAL in the different groups.

	Control Group (*n* = 18)	CI-AKI Group (*n* = 12)	Subclinical CI-AKI Group (*n* = 15)	Control Group vs. CI-AKI	Control Group vs. Subclinical CI-AKI	CI-AKI vs. Subclinical CI-AKI
Serum creatinine (μmol/L)	Median (IQR)	Median (IQR)	Median (IQR)	*p* values	*p* values	*p* values
Baseline	73.6 (67–95)	93.5 (88.3–105.9)	77.0 (74–92)	0.005	0.625	0.014
4th hour	78.2 (71.5–90.4)	104.0 (98.7–112.5)	85.0 (72–100)	<0.001	0.458	0.004
24th hour	79.0 (70.8–92.5)	124.5 (108.5–136.3) *	79.0 (71–96)	<0.001	0.928	<0.001
48th hour	77.5 (66.7–91)	106.0 (91.8–119.5) *	76.5 (69.5–89.5)	0.002	0.884	0.005
GFR (mL/min/1.73 m^2^)						
Baseline	79.8 (72.4–87.3)	69.0 (66.0–78.2)	77.1 (68.8–94.3)	0.020	0.971	0.130
4th hour	76.0 (70.5–85.2)	65.1 (57.7–68.2)	73.4 (62.5–87.3)	<0.001	0.563	0.025
24th hour	77.8 (69.1–90.8)	50.5 (47.7–54.5) *	73.4 (68–91.9)	<0.001	0.914	<0.001
48th hour	77.9 (70.3–94.5)	62.5 (54.1–71.6) *	77.9 (63.1–96.2)	0.010	0.861	0.016
NGAL (ng/mL)						
Baseline	80.3 (66.9–86.2)	97.6 (69.4–127.0)	80.0 (44.4–94.1)	0.072	0.899	0.088
4th hour	76.6 (65.3–87.6)	109.3 (92.1–148.7) *	94.0 (75.5–148.2) *	0.001	0.024	0.354
24th hour	78.0 (66.4–88.5)	131.0 (81.1–240.8) *	100.7 (55–132.2) *	0.005	0.086	0.164

* *p* < 0.05 versus baseline values in the same group. CI-AKI—contrast-induced acute kidney injury; GFR—glomerular filtration rate; IQR—interquartile range.

## Data Availability

The data presented in the current study are available on reasonable request from the corresponding author.
